# Landscape of Push Funding in Antibiotic Research: Current Status and Way Forward

**DOI:** 10.3390/biology12010101

**Published:** 2023-01-09

**Authors:** Himika Wasan, Devendra Singh, K.H. Reeta, Yogendra Kumar Gupta

**Affiliations:** 1 Department of Pharmacology, All India Institute of Medical Sciences, New Delhi 110029, India; 2 Principal Advisor India Strategy Development, Global Antibiotics Research and Development Partnership (GARDP), New Delhi 110016, India

**Keywords:** antibiotics, antimicrobial resistance, push funding, CARB-X, GARDP, ND4BB, BARDA, combating AMR

## Abstract

**Simple Summary:**

Antibiotic resistance has become one of the biggest threats to global health. The occurrence of resistance is a natural phenomenon of bacterial evolution, but their inappropriate use in humans and the environment is hastening the process at a reckless speed. The speed of developing resistance is at par or, in fact, more than the speed of novel antibiotic development. Most of the big pharmaceutical companies have left antibiotic research due to the high risk of failure and poor return on investment. Antibiotic research is mostly carried out by academic institutes and small- and medium-sized enterprises. However, they lack sufficient funds to take the compounds from early-and-mid-stage to clinical trials and market. To make this possible, several government and non-government organizations worldwide have come forward to incentivize research through push funding mechanisms. The positive impact of these mechanisms, which started around a decade ago, is now visible, with fair improvements in research pipelines in the last five years. However, a large landscape of push incentives across the globe and staggered funding across different stages of development make the process complex. Efforts in various forms are now being implemented and proposed to streamline and smoothen push funding mechanisms for reinvigorating antibiotic research.

**Abstract:**

The growing need for effective antibiotics is attributed to the intrinsic ability of bacteria to develop survival mechanisms. The speed at which pathogens develop resistance is at par or even faster than the discovery of newer agents. Due to the enormous cost of developing an antibiotic and poor return on investment, big pharmaceutical companies are stepping out of the antibiotic research field, and the world is now heading towards the silent pandemic of antibiotic resistance. Lack of investment in research has further led to the anemic antibiotic pipeline. To overcome these challenges, various organizations have come forward with push funding to financially assist antibiotic developers. Although push funding has somewhat reinvigorated the dwindled field of antibiotic development by bearing the financial risks of failure, the landscape is still large and staggered. Most of the funding is funneled towards the early stages; however, to carry the promising compounds forward, equal or more funding is required formid- and late-stage research. To some extent, the complexity associated with accessing the funding mechanisms has led to their underutilization. In the present review, we discuss several major push funding mechanisms, issues in their effective utilization, recent strategies adopted, and a way forward to streamline funding in antibiotic research.

## 1. Introduction

Antibiotic resistance is an evolutionary process in which bacteria develop novel survival mechanisms to evade antibiotics. In 2019, antibiotic resistance led to 1.27 million global deaths and is expected to kill approximately 10 million people annually by 2050 [1]. To mitigate the risk of drug-resistant infections, there is a continual need for an optimal and sustainable arsenal of effective antibiotics. Despite antibiotic resistance being the biggest threat to global health, the antibiotic research pipeline remains dry due to various scientific, economic, and regulatory challenges. To tackle these challenges, commendable efforts have been made by various government and non-government organizations in the form of grants and incentives in the last few years. Although, to reinvigorate the antibiotic pipeline, several preclinical and clinical research and development (R&D) programs in the form of push and pull incentives are in place; however, these efforts remain inadequate to address the global health needs. Funding in the form of push incentives to assist in the R&D of antibiotics and pull incentives to facilitate adequate market revenue for novel antibiotic developers are being proposed and implemented. In the present review, we discuss the current status of antibiotic R&D and the trends in push funding. We also discuss the issues with the current push funding mechanisms, recent strategies adopted, and a way forward to effectively utilize these incentives in a targeted and coordinated approach.

## 2. Current Status of Antibiotic Research and Development

The discussion over the early blooming era of antibiotics cannot be completed without mentioning Paul Ehrlich and Alexander Fleming. Ehrlich’s idea of magic bullets for syphilis in the form of salvarsan and Fleming’s serendipitous discovery of penicillin were prototypical, as they set a way forward for the discovery of many newer antibiotics. This led to the beginning of the golden era of antibiotics between the 1950s and the 1970s, when a large number of novel classes of antibiotics were discovered [2]. Simultaneously, resistance to these new antibiotics also started developing. However, the development of novel antibiotics after that period slowed down as the existing arsenal of antibiotics was effective, and by the early 1980s, the focus of pharmaceutical companies, based on public health needs, shifted towards more lucrative fields such as cancer and lifestyle diseases. The discovery void started to appear from early 2000 to 2010, when the majority of the big pharmaceuticals left the antibiotic market.

Since then, the scientific community in academia, small companies, and a few large pharmaceutical companies have been putting their efforts into the R&D of novel antibiotics. However, the investment is no longer sufficient to move the compounds from the early stages to approval. Further, after approval, the market potential is small due to low net present value (NPV, a value calculated based on ultimate costs and revenue) since, in most cases, the antibiotics are prescribed for a short duration with the best possible use of alternative generic antibiotics. Therefore, despite the high societal monetary benefits of new antibiotics (ranging from 486 million USD to 12 billion USD), the NPV of novel antibiotics remains low (negative) with an average of −50 million USD, in contrast to +1.15 billion USD for a new musculoskeletal drug [3]. Hence, many big pharmaceutical companies have left and are leaving the field of antibiotic research to invest in other lucrative areas. Recent examples are the exit of Novartis and Sanofi in 2018 from antibiotic R&D, and as of now, only six, including Pfizer, Roche, Otsuka, Merck, Shionogi, and GlaxoSmithKline have visible programs [4]. Until September 2021, there were 217 antibacterial products or programs in preclinical phases, with 84% of the research activities being carried out by micro-, small- and medium-sized developers and only 16% by large developers [5].

This disconnect between costly antibiotic development and low NPV stresses the need for financial incentives that can either decrease the cost of R&D or increase the market revenues. Therefore, several global organizations have put forward funding strategies to lower the cost of developing an antibiotic, directly or indirectly, by cutting down the risk of failures, as they cover both successful and unsuccessful projects. Of the major financial incentives in the field of antibiotic R&D, 71% are strictly push incentives funding the development of novel antibiotics [6]. For instance, the flourishing preclinical antibiotic pipeline in Europe (52%) and America (35%) can be attributed to the proactive approach of government and non-government philanthropic organizations in these continents [5]. Although these strategies showed a positive impact on NPV, they are insufficient as they do not cover the revenue generation after antibiotic approval and are inadequate alone to recuperate the dried antibiotic arena [6]. Therefore, equal attention to market sustainability through pull incentives is needed after antibiotic approval to mitigate the market failure challenges, as evidenced by the bankruptcy of Achaogen, an SME that developed plazomicin. Achaogen was unable to sustain the market of plazomicin despite being push funded from initial stages to clinical trials by Wellcome Trust, the National Institute of Health (NIH), and the Biomedical Advanced Research and Development Authority (BARDA) [7,8]. In the following sections, we review the trends of push funding across various countries.

## 3. Trends in Push Funding

In the last decades, several high-profile panels and working groups across the globe gained political momentum to incentivize antibiotic R&D. This led to the development and implementation of various national as well as global programs in the form of push incentives to subsidize the developmental cost of antibiotics. In this section, we discuss several major country-level and global initiatives taken by the United States of America (US), the European Union (EU), and the United Kingdom (UK), the current dominating players in research, in incentivizing antibiotic R&D through push funding.

### 3.1. Country-Level Initiatives

#### 3.1.1. US-Based National Initiatives

- National Institute of Allergy and Infectious Diseases (NIAID)

In 2008, NIAID, a center of the NIH under the Department of Health and Human Services (DHHS), took a lead role in addressing antimicrobial resistance (AMR) through funding grants, contracts, and other mechanisms. NIAID supports basic, translational, and clinical research portfolios to pave the way for innovative solutions for preventing, diagnosing, and treating drug-resistant infections. From 2008 to 2022, the NIH supported funding of 4.5 billion USD to tackle AMR [9]. The basic research portfolio focuses on understanding the resistance mechanisms, delineating contributors to bacterial virulence, and identifying newer targets and potential approaches for diagnostics, vaccines, and therapeutics. A translational research portfolio aids in transforming basic research findings into applications for the development of therapeutic and diagnostic products. Through the Centers of Excellence for Translational Research program, NIAID is supporting translational research for the development of novel therapeutics for drug-resistant infections [10]. The grants to accomplish basic and translational research are awarded for 2–5 years to academia and small businesses in the category of R01 NIH Research Project Grant Program, R03 NIH Small Grant Program, R41/R42 Small Business Technology Transfer (STTR; for collaboration between small business concerns and research institutions) and R43/R44 Small Business Innovative Research (SBIR) [11].

NIAID also offers a variety of preclinical and clinical research resources through its comprehensive set of product development services and research tools to facilitate the development of next-generation diagnostics, vaccines, and therapeutics. These include in vitro and animal model screening tools, and Biodefense and Emerging Infections Research (BEI) resources repository, which provides free-of-cost microorganisms and reagents to microbiology and infectious disease researchers, and Antibacterial Resistance Leadership Group (ARLG) virtual biorepository that provides clinical study isolates and well-characterized gram-positive and gram-negative bacterial isolates to evaluate mechanisms of resistance and to develop diagnostics and therapeutics [12,13]. The NIH has also developed the open-access National Database of Antibiotic Resistant Organisms maintained by the National Center for Biotechnology Information, which contains genomic data on bacterial resistance, which is accessible to scientists all over the world, to enhance the understanding of resistance mechanisms [14]. The resources and funding provided by NIAID to academia and SMEs for carrying out antibiotic R&D are substantial and appreciable.

- Food and Drug Administration (FDA)

Since the announcement of a national strategy for Combating Antibiotic-Resistant Bacteria (CARB) in 2014, the FDA has played an integral role in addressing this global threat. FDA facilitates efficient product development, responsible use, active surveillance, and advancing regulatory science for translating breakthrough discoveries into innovative, safe, and effective applicable products [15]. The FDA, through its ‘Office of Infectious Diseases Research Activities, assists antimicrobial regulatory science research funding by facilitating the development of new antibiotics and advancing clinical trial designs. In the fiscal year 2017–2021, research focused on eight priority areas, including animal model development for infections caused by *Acinetobacter baumannii* or *Pseudomonas aeruginosa*; understanding antibacterial market; understanding human gut and lung microbiomes; developing patient-reported outcomes for non-cystic fibrosis bronchiectasis; guidelines for infectious diseases; science of drug susceptibility testing; developing patient-reported outcomes for non-tuberculous mycobacterial disease and coccidioidomycosis; and evaluating the impact of extended infusion of β-lactam antibiotics [16]. The funding was given in the form of grants, interagency agreements, and contracts through the FDA’s Broad Agency Announcements for the Advanced Research and Development of Regulatory Science [16]. For the fiscal year 2023, five requests for proposals have been announced todate in the area of therapeutics and diagnostics, with a total funding of 3 million USD [17]. The FDA, in collaboration with the Center for Disease Control and Prevention (CDC), developed the Antibiotic Resistance Isolate Bank (AR Isolate Bank) in 2015, which contains a repository of resistant bacterial and yeast isolates derived from the community and healthcare-associated infections, food-borne illnesses, and sexually transmitted diseases. The well-characterized isolate panels are used in the development of novel antibiotics, diagnostics, and for studying resistance mechanisms. These isolates are made available free of charge for research to the requesting institutes. Until April 2022, the AR Isolate Bank has shipped more than 269,000 isolates in 7000 panels to various approved institutes, including academic institutes, pharmaceutical companies, and clinical and health laboratories [18,19]. The FDA Antimicrobial Resistance Taskforce is collaborating with various government and other organizations to develop approaches to detect, prevent and limit the development of resistance. Additionally, through its research activities, the FDA facilitates the development of novel agents to fight drug-resistant infections.

- Biomedical Advanced Research and Development Authority (BARDA)

After the anthrax attack of 2001, the US President passed the Project BioShield Act in 2004 to improve medical countermeasures against the chemical, biological, radiological, and nuclear (CBRN) attacks for protection of the Americans [20]. In 2006, the Pandemic and All-Hazards Preparedness Act (PAHPA) was passed “to improve the Nation’s public health and medical preparedness and response capabilities for emergencies, whether deliberate, accidental, or natural.” Subsequently, PAHPA led to the development of BARDA as a component of DHHS with a mission to secure the nation from mass public health emergencies, including CBRN threats, pandemic influenza, and emerging infectious diseases [21]. In 2010, BARDA initiated a program to address AMR and established various public-private partnerships to develop novel antibiotics and diagnostics platforms. From 2010–2019, BARDA awarded 959 million USD in the form of grants, agreements, and contracts to develop antibiotics [22]. BARDA, in 2013, invested 403 million USD in four big pharmaceutical companies (GSK, AstraZeneca, the Medicines Company, and Hoffmann-La Roche) involved in the development of seven antibiotic candidates through Other Transaction Authority (OTA), an innovative and flexible contracting channel that helps BARDA to enter international collaborations. BARDA also supported the development of Combating Antibiotic Resistant Bacteria Biopharmaceutical Accelerator (CARB-X) in 2016, an international non-profit organization involved in early preclinical research and Phase-1 trials. Additionally, BARDA announced a prize of 20 million USD in collaboration with the NIH for the development of innovative diagnostic tools that can distinguish viral and bacterial infections and can characterize antibiotic-resistant bacteria under the AMR Diagnostic Challenge [23]. In 2020, BARDA, in collaboration with the NIH, awarded 19 million USD to Visby Medical for developing rapid diagnostics to detect and perform susceptibility testing of microorganism that causes gonorrhea [24]. Although it does not fund basic science, BARDA supports companies developing antibiotics from the preclinical stage to marketing approval through CARB-X and its advanced development support program. BARDA’s financial support in late-stage clinical development led to the approval of many novel antibiotics, including meropenem-vaborbactam [25], ceftazidime-avibactam [26], plazomicin [27], and eravacycline [28].

- Department of Defense (DOD)

The DOD also funds and conducts research for the development of novel treatments for antibiotic-resistant infections. Since 2012, the DOD has awarded approximate funding of 271 million USD through Defense Threat Reduction Agency (178 million USD funding for 21 projects since 2012), U.S. Army Medical Research and Materiel Command (66.2 million USD for 50 projects since 2012), Walter Reed Army Institute of Research (10 million USD since 2016 for 16 projects) and Defense Advanced Research Projects Agency (17.1 million USD for five years since 2014) [22]. In 2021, Defense Threat Reduction Agency awarded 75 million USD to the University of Florida for the development of novel antibiotics [29].

The above-discussed funding agencies are the major contributors to push funding in the US and have supported many preclinical and clinical antibiotic research projects. Since most of the projects have been aggressively active in the last decade, the actual impact of the funding will be visible only in the coming years. Nevertheless, the status of antibiotic research is gradually improving.

#### 3.1.2. EU-Based National Initiatives

- Innovative Medicine Initiative-New Drugs for Bad Bugs (IMI-ND4BB)

European Technology Platform (ETP) on Innovative Medicines was the foundation stone for the development of the Innovative Medicine Initiative (IMI), which aims to boost drug development in Europe. The strategic research agenda was developed by ETP (2005–2009) by involving various stakeholders from the pharmaceutical sector, which led to the formation of IMI in 2007. IMI is a public-private partnership between the European Commission (EC) and the European Federation of Pharmaceutical Industries and Associations (EFPIA). The first project, IMI1 (2008–2013), aimed to improve the efficiency and effectiveness of the drug development process with a long-term goal of effective and safe innovative medicine by the pharmaceutical sector with a total budget of 2 billion euros. The EU’s Seventh Framework Programme (FP7) contributed 1 billion euros, and the rest was contributed by EFPIA and its member companies. This funding supported many research, education, and training projects in the field of oncology, neurology, diabetes, infections, etc., by research centers, universities, SMEs, patient groups, and regulators in EU member states and FP7-associated countries [30,31]. IMI also helped in the creation of a European platform to tackle AMR and to discover new antibiotics through the New Drugs for Bad Bugs (ND4BB) program under its 2011 action plan on AMR [32]. The ND4BB program received funding of 650 million euros to develop novel antibiotics and to cover the R&D pipeline from basic sciences to clinical trials and market economics under its eight projects. These projects include TRANSLOCATION (29.7 million euros), ENABLE (100.7 million euros), COMBACTE-NET (212.5 million euros), COMBACTE-CARE (85.1 million euros), COMBACTE-MAGNET (89.5 million euros), COMBACTE-CDI (4.1 million euros), iABC (56.2 million euros) and DRIVE-AB (10.9 million euros). TRANSLOCATION project of ND4BB funds the basic science projects addressing the challenges of antibiotic resistance through understanding potential barriers to antibiotic penetration and efflux mechanisms, primarily among gram-negative pathogens. ENABLE platform helps in advancing the molecule from the testing and optimization stage to early clinical research in universities and SMEs. The COMBACTE group of projects helps in the clinical developmental phases by developing strong research, laboratory, and clinical networks through COMBACTE-NET. COMBACTE-CARE focuses on the clinical development of antibiotics for carbapenem-resistant *enterobacteriaceae* (CRE), COMBACTE-MAGNET focuses on developing preventive and therapeutic treatments for life-threatening hospital-acquired infections, and lastly, COMBACTE-CDI focuses on developing antibiotics for *Clostridium difficile* infection (CDI). The iABC project leads the clinical development of inhaled antibiotics for bronchiectasis and cystic fibrosis patients. The main agenda of the DRIVE-AB project is equitable and sustainable access to antibiotics. DRIVE-AB safeguards the continued interest of product developers in investing in novel antibiotics by developing commercial models that will incentivize and reward new developments [33]. These projects cover almost the entire antibiotic pipeline, from molecular synthesis to the clinical phases. They have brought together experts from various domains, including academia, industries, SMEs, and regulatory bodies, thereby facilitating collaboration and risk sharing [33]. ND4BB placed Europe atthe center of the global fight against antibiotic resistance. The first phase of IMI, IMI1, was completed in 2013 and then moved to the second phase, IMI2, under Horizon 2020. IMI2 carried forward the AMR agenda of IMI1 with the aim of building Europe as a global leader inhealthcare solutions [34].

- Innovfin Infectious Disease Facility (IDFF)

The IDFF is another financing facility launched jointly by the European Commission and the European Investment Bank Group. It was launched in 2014 with the aim of financing late-stage projects. The facility provides loans in the range of 7.5 million to 75 million euros to SMEs, large pharmaceuticals, and other research facilities such as universities or non-profit organizations working in the area of innovative therapeutics and diagnostics for infectious diseases. The project funds 28 EU member states and 17 other countries associated with Horizon 2020. The funding is based on standard debt to risk-sharing instruments, giving an immediate kick-start for the projects [35].

#### 3.1.3. UK-Based National Initiatives

In the UK, the Medical Research Council (MRC) and Biotechnology & Biological Sciences Research Council (BBSRC) under the UK Research and Innovation councils (UKRI), and the National Institute for Health and Care Research (NIHR) under the Department of Health and Social Care (DHSC), play a key role in antibiotic research at the country level. To boost antibiotic research and innovation, MRC established the UK AMR Funders’ Forum (AMRFF) in 2014,bringing together 21 research funders, including UKRI and various other government departments. Four thematic areas were identified by the funders to target their investments, including understanding resistant bacteria, development of diagnostics and therapeutics, understanding real-world interactions, and investigations on the behavioral impact of public and professional organizations. MRC also led to the initiation of the AMR Cross Council Initiative called Tackling AMR (co-funded by MRC, BBSRC, and Natural Environment Research Council; NERC under UKRI). Since 2014, the UK government has invested more than 360 million pounds in antibiotic research and innovation coordinated through AMRFF at the country level and for global research [36].

- Medical Research Council (MRC) and National Institute for Health and Care Research (NIHR)

MRC funds research activities through its five grant programs, namely research, programme, partnerships, new investigator, and MRC Industry collaboration framework [37]. The funding is provided to higher educational institutes, independent research organizations, National Health Service (NHS) bodies, public sector research establishments, MRC institutes, MRC units, partnership institutes, and institutes and units funded by other research councils [38]. The NIHR is another England-centric UK government body funded by the DHSC that focuses on translational and clinical research in partnership with the NHS, universities, local government bodies, and research organizations [39]. It provides a range of support to universities and life sciences organizations through their biomedical research centers (BRCs) that partner with universities with an aim to provide bench-to-bedside medicines [40]. In 2022, the NIHR awarded 800 million pounds to 20 BRCs associated with universities for developing new treatments, technologies, and diagnostics [41]. Of these, four universities, including Cambridge, Imperial, Oxford, and Southampton BRCs, work on antimicrobial and infection programs.

### 3.2. Global Initiatives

#### 3.2.1. US-Based Global Initiatives

- Antibacterial Resistance Leadership Group (ARLG)

In 2013, the NIAID launched ARLG to advance clinical research to address AMR. Different types of clinical studies conducted by ARLG include the evaluation of diagnostics in clinical settings, clinical testing of new drugs for drug-resistant gram-negative infections, and optimization of treatment regimens to prevent drug-resistant infections. The long-term goals of the ARLG Committee are to identify, design, and implement transformational clinical trials to improve outcomes of extensively drug-resistant infections and minimize resistance [42]. By the end of 2019, the NIAID renewed funding of up to 102.5 million USD over seven years for ARLG. The ARLG research team is now collaborating with 19 countries and has initiated around 40 clinical studies across 130 sites involving more than 20,000 patients for clinical testing of therapeutics for gram-negative and gram-positive infections as well as for the development of rapid point-of-care diagnostics [43].

- Combating Antibiotic-Resistant Bacteria Biopharmaceutical Accelerator (CARB-X)

CARB-X is a non-profit global organization established in 2016, which provides non-dilutive funding for early stages of development, from hit to lead to Phase 1 trials for therapeutics (traditional/non-traditional antibiotics and vaccines) and feasibility determination through validation and verification for diagnostics. Headquartered in Boston, CARB-X is funded by BARDA, the Wellcome Trust, Germany’s Federal Ministry of Education and Research, UK’s Global Health Security’s Global AMR Innovation Fund (GAMRIF), Bill and Melinda Gates Foundation, and the NIAID. The NIAID provides in-kind services such as pre-clinical services to CARB-X-funded projects valued at 50 million USD [44,45,46]. From 2016–2022, CARB-X received 1163 applications from 39 countries and has funded 92 projects with a total investment of 480 million USD, of which 12 have graduated, meaning they achieved their final milestones of preclinical and Phase 1 studies and are now in the later stages of clinical development [47]. There are 39 active projects, including 32 therapeutic projects and seven diagnostic projects [48]. To further strengthen the growing portfolio of early-stage research, CARB-X partnered with nine accelerators across five countries, who provide technical, scientific, and business support to CARB-X product developers [49]. In 2021, CARB-X announced the Stewardship and Access Plan (SAP) for CARB-X-funded companies. According to the Plan, CARB-X-funded developers are obligated to develop SAP as soon as the product enters the pivotal trial phases, whichis generally Phase 3 trials or equivalent for diagnostics. In the plan, the product developers will outline the strategies to be deployed for responsible use after approval and the access plans for equitable access across countries [50]. The CARB-X research focus is based on the WHO and CDC priority list of bacteria responsible for serious infections. The call for funding is open to all public and private organizations across the globe.

#### 3.2.2. EU-Based Global Initiatives

- Joint Programming Initiative on Antimicrobial Resistance (JPIAMR)

JPIAMR is a global collaborative platform of 29 member countries and the European Commission working together to combat AMR with a One Health approach. JPIAMR is a European initiative established in 2011 with the objective of overcoming the fragmented AMR research system. The first joint research call for funding on novel therapies was published in 2014, and until now, 13 translational joint calls have been coordinated. JPIAMR is currently funding 99 projects and 38 networks with a total investment of approximately 125 million euros to fund research and innovation in six key research priority areas, including surveillance, diagnostics, therapeutics, interventions, environment, and transmission [51]. JPIAMR does not pool individual member funds; rather, the funding for R&D is paid through the national research agency. The G20, G7, and EU recognize JPIAMR as a key mechanism enabling collaboration and coordination of activities in the field of AMR in Europe and around the world. To support and coordinate transformative research, JPIAMR will regularly fund through national research agencies for proposals focusing on research and innovations under its six priority areas. JPIAMR will also support networking and partnerships among research communities of member countries to reduce the chances of effort duplication [52]. The research grants are normally awarded for a period of 36 months to a consortium of 3–6 investigators from a minimum of three participating countries to work on a joint research project. The funding is mainly directed to academic and research institutes to carry out early-phase research with a total average funding of 1.2–1.5 million euros per project, which is too low to attract industry partners [53]. In the updated Strategic Research and Innovation Agenda 2021, JPIAMR recognized the need to support academia-industry collaboration to streamline the process oftranslating positive research outcomes into products, services, and policies [54].

- European and Developing Countries Clinical Trials Partnership (EDCTP)

The EDCTP, launched in 2003 and a second phase (EDCTP2) in 2014 (2014–2024), is an evolving public-private partnership between 14 European and 16 sub-Saharan African countries in collaboration with the EU and the pharmaceutical industries to advance the development of medical interventions in the area of infectious diseases [55]. The mission of EDCTP is to strengthen research capacity and medical interventions to identify, cure and prevent poverty-related infectious diseases. They primarily support Phase 2 and Phase 3 clinical trials in the area of diagnostics, drugs, and vaccines for HIV/AIDS, malaria, tuberculosis, diarrheal diseases, respiratory infections, and emerging/re-emerging infections [56]. As of December 2021, EDCPT2 had granted a total funding of 814.3 million euros for 431 projects targeting clinical research, building research infrastructure, and fellowships for the career development of Africa-based scientists. It also aims to strengthen national regulatory capacity through regulatory harmonization across participating countries. EDCTP2 also supports global coordination with active involvement from other countries, such as the US. The EDCTP2, under the Horizon 2020 program of Europe, took proposal calls until 2020. The EDCTP2 has now moved to the next phase (2021–2025) to manage the current projects and has stopped funding for new projects. The EDCTP2 has now transitioned, in the year 2022, into Global Health EDCTP3 Joint Undertaking (GH EDCTP3 JU). It is a partnership between the EU and EDCTP focusing on ‘Creating a sustainable clinical trial network for infectious diseases in sub-Saharan Africa’ and ‘Strengthening regulatory capacity for supporting the conduct of clinical trials.’ The total budget for funding will be approximately 1.6 billion euros [57,58].

- Global Antibiotic Research and Development Partnership (GARDP)

The GARDP is a not-for-profit organization created in 2016 by World Health Organization (WHO) and the Drugs for Neglected Diseases initiative (DNDi) to deliver a global action plan on AMR. GARDP became an independent organization in 2019 and is designed to promote innovations to tackle AMR. GARDP harnesses experience and insight from leaders across various public and private sectors, including government, the United Nations, industry, academia, and civil society. They also connect the leadership to provide directions and support for the development of novel antibiotic treatments. Their mission is to make antibiotics accessible across the globe for every person in need [59]. Their R&D portfolio focuses on five programs, including (i) discovery and exploratory, (ii) children’s antibiotics, (iii) sexually transmitted infections, (iv) serious bacterial infections, and (v) access to antibiotics. Of these five programs, the first four programs focus on early and late-stage R&D, and the last program is more towards access to generic and novel antibiotics [59].

The discovery and exploratory program focus on three interrelated activities, including exploratory research to identify novel targets, hit identification, screening, and development of enabling technology and facility platforms. To achieve this, GARDP established the AMR screening consortium in 2018 with three Japanese pharmaceutical companies comprising Eisai, Takeda, and Daiichi Sankyo to access their compounds library for the screening of antibiotic activity. They screened the compound libraries at Institut Pasteur Korea, an institute focused on researching infectious diseases. GARDP also partnered with the US non-profit translational research institute, Calibr at Scripps Research for its ReFRAME compound library and Germany’s Helmholtz-Institute for Pharmaceutical Research Saarland for its natural compound library to be tested at the University of Queensland’s (UQ) Community for Open Antimicrobial Drug Discovery (CO-ADD) to discover novel compounds and combinations for activity against the WHO priority pathogens. Till now, GARDP has screened over 100,000 compounds and more than 10 chemical series for antibiotic activity, of which four have moved to hit identification stages [60]. Under the children’s antibiotic program, GARDP partnered with Penta, the pediatric infectious diseases research network in Italy, to develop global children’s antibiotic platform to accelerate antibiotic development with a focus on neonatal sepsis and pneumonia [61]. Further, in collaboration with Venatorx Pharmaceuticals, GARDP is developing a novel combination of cefepime-taniborbactam against WHO critical priority pathogens (carbapenem-resistant *Enterobacteriaceae*; and carbapenem-resistant *Pseudomonas aeruginosa*;) and will expedite the pediatric trials once approved. They are also putting efforts to make existing antibiotics suitable for pediatric use [62]. In association with Entasis Therapeutic, Phase 3 clinical trials of zoliflodacin, a novel antibiotic being developed for gonorrhea, are being conducted [63]. In the serious bacterial infection program, GARDP has partnered with Venatorx Pharmaceuticals for Phase 3 trials to co-develop cefepime-taniborbactam. An agreement has also been signed with BioVersys, a Swiss biopharmaceutical company, to explore opportunities for R&D of antibiotics required to treat serious bacterial infections [64,65]. GARDP is a pipeline coordinator and is coordinating antibiotic R&D activities ranging from the screening stage to bringing antibiotics to the market. In an effort to bring the scientific community together, GARDP launched REVIVE platform in 2018. This is an outreach activity that ensures knowledge dissemination between academic, clinical, and industrial researchers, which will help in improving, accelerating, and streamlining the efforts across the antibiotic R&D field. The platform connects budding researchers with established and retired researchers and developers to facilitate knowledge exchange through various educational and collaborative activities [66].

#### 3.2.3. UK-Based Global Initiatives

The UK plays a leading role in the global efforts to combat AMR and has committed 464.4 million pounds in the period of 2016–2022. There are four major funders in the UK to fund antimicrobial research, including three government departments comprising the Department for International Development (DFID), the Department for Business, and the Energy and Industrial Strategy (BEIS) through UKRI and the Department for Health and Social Care (DHSC) through the UK government’s Official Development Assistance (ODA) budget and one non-government organization, Wellcome [67].

- Department for International Development (DFID)

The DFID has invested approximately 161 million pounds in a five-year period (2017–2021) in AMR-relevant research through non-profit product development partnerships to bring together various public-private stakeholders with aggregated funding. Examples include product development partnerships with Foundation for Innovative New Diagnostics (FIND), GARDP, Meningitis Vaccine Project (MVP), etc. Their research focuses on product development, such as vaccines, diagnostics, drugs, surveillance, etc. [67].

- Department for Business, Energy, and Industrial Strategy (BEIS)

The BEIS, through Global Challenge Research Funds (GCRF) and Newton Funds, has invested approximately 33.8 million pounds through global AMR calls (2016–2021), which are administered through MRC’s Cross Research Council Initiative. GCRF was launched in 2015 with the aim of achieving UN Sustainable Development Goals by addressing global issues faced by developing countries through funding research and innovation. In the field of AMR, GCRF has funded 9.7 million pounds under Cross Council Initiative: Tackling AMR program, 3 million pounds under MRC AMR Target discovery and validation, and 0.21 million pounds through Arts and Humanities Research Council (AHRC)-led grant from AMR in the Built and Indoor Environment. The Newton Fund, launched in 2014, focuses on interdisciplinary partnerships with 16 countries in Asia, Africa, and Latin America to promote economic development and welfare through matched funding. In the field of AMR, their focus is on diagnostics and therapeutics under the One Health umbrella. In 2016, under UK-China AMR Initiative Partnership, a 4.5 million pounds Newton fund supported six interdisciplinary research partnerships between UK and China with matched funding from the National Natural Science Foundation of China to foster collaboration across borders to tackle AMR [68]. In 2017, under Newton funds, the UK committed a matched funding of 8 million pounds to create the UK-China AMR Centre Partnerships Hubs. Similarly, in India, under a Newton fund-UK-India partnership, a 6.5 million pounds Newton fund was committed in 2017 to tackle AMR through collaborative and interdisciplinary research partnerships [67].

- Department for Health and Social Care (DHSC)

The DHSC, through Global Health Security’s Global AMR Innovation Fund (GAMRIF), invested 57 million pounds (2016–2019) that support high-quality early-stage R&D in underfunded and neglected disease areas of AMR to diagnose, prevent and treat drug-resistant infections in resource-limited settings. GAMRIF’s research portfolio keeps a focus on the One Health approach, investing together in research across human, animal, and environment; and until 2019, had supported seven work packages including UK-China collaboration, CARB-X, InnoVet-AMR with International Development Research Centre, UK-Argentina-tools to tackle AMR in the environment, innovation in diagnostics with the FIND, new treatment for drug-resistant gonorrhea infections with GARDP and vaccine innovation with BactiVac Network [36].

- Wellcome

Under the non-government organizations, Wellcome, a global charitable foundation, was established in 1936 with the aim of supporting science to solve urgent health issues faced globally. Wellcome takes into account the three biggest challenges faced by humanity, that is, mental health, infectious diseases, and climate change, and funds curiosity-driven research in these areas [69]. Under the infectious disease program, their goal is to understand disease etiology and the impact of the disease through surveillance, support R&D from early-stage to clinical trial stages and ensure an equitable regulatory environment. These goals can be achieved through funding, partnerships, advocacy, and collaborative work with various communities [70]. The Wellcome Foundation has invested 175 million pounds (125 million pounds in CARB-X and 50 million pounds in AMR Action Fund) to fund early-stage antibiotic R&D and to help biotech companies in carrying out complex and expensive clinical trial R&D stages [71].

### 3.3. Pharmaceutical Industry Initiatives

#### 3.3.1. Replenishing and Enabling the Pipeline for Anti-Infective Resistance (REPAIR) Impact Fund

The REPAIR Impact Fund, launched in 2018, is the Novo Holdings initiative that funds early-stage development, between lead optimization and Phase 1 trials, for drug-resistant infections identified by WHO and CDC as priority pathogens. With a total funding of 165 million USD, it aims to invest 20–40 million USD per year in about 20 projects run by start-ups, early-stage companies, and corporate spinouts of Europe and the US for 3–5 years [72]. REPAIR Impact fund has also committed to keeping some capital reserves to support Phase 2 clinical trials for its portfolio companies. Until now, it has invested in ten anti-infective companies developing a range of antibiotics and vaccines [73].

#### 3.3.2. AMR Action Fund

The AMR Action Fund, an initiative of the International Federation of Pharmaceutical Manufacturers & Associations (IFPMA; international body representing the R&D pharmaceutical industry), was launched together by 20 leading biopharmaceutical companies in 2020 [71]. The aim of the AMR Action Fund is to fund pharmaceutical companies to bring 2 to 4 potentially lifesaving antimicrobial therapeutics by 2030 and to create a sustainable ecosystem of investment and innovation. The companies, so far, have raised a funding of 1 billion USD to fund effective traditional and non-traditional agents at various clinical stages of development [74]. The Fund makes equity investments in SMEs developing therapeutics against WHO and CDC priority pathogens. The portfolio companies funded by AMR Action Fund have to develop access plans for the broader registration of their product. In addition, the portfolio companies are expected to be a member of the AMR Industry Alliance and will report their progress toward industry commitments related to research, access, appropriate use, and manufacturing. The commitment also includes advancing stewardship, involvement in surveillance programs by means of data sharing with healthcare professionals and public health organizations, as well as active engagement with diagnostic companies to ensure appropriate use. The Fund aims to bring together the wide-ranging alliance of industry and non-industry stakeholders, including development banks, multilateral organizations, and philanthropies, that can encourage the government to create conditions for sustainable investments in the antibiotic market [74].

## 4. Challenges and the Way Forward

The efforts put globally in the form of push incentives to boost and furnish the antibiotic R&D pipeline are commendable and valuable. Governments and regulatory bodies of big economies started many initiatives, mostly in the last decade (Table 1). A huge amount of funding for antibiotic R&D have now been floated through various national and global initiatives.

There is, nevertheless, an imbalance in the distribution of funds as a majority of the funding is funneled towards early-stage research, and various mechanisms of funding exist for academic and research institutes. Typically, if we look at the drug discovery pipeline, there are mainly three stages—early-stage carrying out basic science research including technology readiness levels (TRL) 1 and 2; mid-stage or preclinical research with TRL 3–5 (3: hit to lead identification, 4: lead optimization and 5: preclinical development) and late-stage or clinical stage with TRL 6–8 corresponding to Phase 1, Phase 2 and Phase 3 clinical trials [53]. In the field of antibiotic research, the early phase is primarily carried out by academic and research institutes. These are largely funded by public and philanthropic organizations, and they carry a high risk of failure. In the mid-stage, when there is a ray of hope, SMEs try to take the promising compounds forward from early-stage discovery to mid-stage and attempt to bring the compounds into clinical trials. Here the plethora of problems start as a lot of funds are required to run clinical trials, and even if the SMEs manage to accomplish this, they go into crisis at later stages in the market due to poor economic incentives. If we look at the antibacterial preclinical pipeline by WHO, as of September 2021, there were 217 compounds in preclinical stages, with more than 80% of the research carried out by SMEs in Europe and the US. More than 85% of compounds are in mid-stage development in TRL 4 & 5 and only 1% at the Investigational New Drug (IND) stage [5,75]. A lot of funding agencies are now streamlining their funds toward mid-stage development. However, on the contrary, SMEs receive only 20% of the public funding and eventually run out of business [76]. The reason for the difficulty in the outreach of funding to SMEs can be attributed to the complex and lengthy process of application and a lack of clarity on the ownership of intellectual property rights. This is the point of attrition when most of the potential compounds lose their future. Nevertheless, early-stage funding is important for replenishing the anemic pipeline as the success rate at this stage is low, but it is equally important to appropriately allocate funds to SMEs for transitioning towards clinical trial phases.

To address these issues of SMEs, the EU launched Biotech companies from Europe innovating in Anti-Microbial resistance research (BEAM) Alliance in 2015 to raise the voice of SMEs involved in innovative antibiotic research. Currently, the BEAM alliance is a group of 69 SMEs spanned across 16 European countries with 84 products in preclinical research, 26 in clinical development, and two in the market [77]. The BEAM Alliance maintains and promotes awareness about innovation driven by SMEs and sensitizes policy makers to incentivize these SMEs in simple and accessible ways to make R&D sustainable [78]. Currently, amongst non-dilutive funding, the Eureka Eurostars programme, European Innovation Council (EIC) Pathfinder, and EIC Accelerator Open is supporting SMEs, and amongst long-term engaging institutions and programs, BARDA, CARB-X, IDFF, GARDP, and AMR Action Fund are playing key roles. Despite these efforts, there are still issues in getting funding as SMEs are small, widely distributed, and resource-limited and usually have no public affairs departments for communication with funding agencies. Therefore, the funding mechanisms are required to be simplified, and the governing body should clearly identify the beneficiary and make funding readily available and accessible.

Another issue is the staggered funding mechanisms that can lead to a significant increase in the risk of effort duplication. There should be a global consortium of researchers as well as funding mechanisms to look after antibiotic R&D. As rightly mentioned by Ardal et al. (2018), there is a need for a Grant Incentives Framework to address the scientific and economic bottlenecks of antibiotic R&D [53]. All the existing funding mechanisms should be channeled in such a way that they could be effectively utilized to create a robust pipeline. These authors suggested categorization of funding into early-stage, mid-stage, and clinical-stage grants of 3–5 years duration. Early-stage grants could focus on basic science research, mainly in academic and research institutes, with a broad scope covering research on priority pathogens as well as in other areas of the antibiotic field. The mid-stage grants could be focused on priority pathogens and majorlycover SMEs to help them in taking their products forward toward later stages. The funding for clinical trials could be towards SMEs and other developers in a way that public health needs are appropriately targeted, and the large investment risks in clinical trials would be reduced. They also proposed ‘Priority grants’ that would strictly focus on WHO and CDC priority pathogens responsible for emerging or immediate threats. These grants are aimed toward long-term funding along the whole drug discovery pipeline, starting from TRL1 to TRL8. In this way, the present push incentives may focus antibiotic R&D toward unmet health needs [53].

GARDP, a pipeline coordinator, has put praiseworthy efforts into developing novel treatments from scratch that is from basic science research to late-stage trials to regulatory approvals and making them accessible globally. Similarly, ND4BB’s eight programs coordinate research activities from early-stage research to clinical trials, as well as making them accessible through its DRIVE-AB project. CARB-X is another strategic initiative to fund early- and mid-stage research, which after graduation are considered by BARDA for further funding of clinical trials. One of the examples under the CARB-X-BARDA portfolio is the development of VE303, a live biotherapeutic product of the Vedanta Biosciences being developed for treating patients at high risk of recurrent *Clostridioides difficile* infections. The early development support was provided by CARB-X with a research grant of 5.4 million USD in 2017, and later, the BARDA’s advanced development support funded a contract of up to 76.9 million USD in 2020 to support Phase 3 trials [79,80].

Despite all these efforts, a need for a single global governing entity that could look after and coordinate various national and international efforts was felt. The G20 leaders called for “a new international R&D Collaboration Hub to maximize the impact of existing and new antimicrobial basic and clinical research initiatives” at a 2017 G20 summit in Hamburg, Germany. This led to the launch of the Global Antimicrobial Resistance (AMR) Research and Development (R&D) Hub in 2018 [81,82]. The Global AMR R&D hub is not a funding mechanism but is a central platform that continuously collects and presents information on global investments in antibiotic R&D to help inform government and non-government funders in priority settings and decision-making for resource allocation [83]. This hub is open to all government and non-government funding agencies in G20 as well as non-G20 countries investing in antibiotic R&D. Its Dynamic Dashboard continuously updates information on AMR R&D investments and activities across One Health continuum and currently presents information of more than 12,000 projects funded by 222 funders with an investment of more than 10 billion USD [84]. This initiative is a major step in establishing internationally agreed priorities and will help in coordinating and streamlining existing as well as new initiatives.

Similar to the Global AMR R&D hub, we propose to the policymakers the consideration of a country-wise centralized database that should have information on completed and ongoing research projects in various institutions of the country. There should be a centralized funding mechanism, and it will be better to have a single funding agency looking after the antibiotic research being conducted in the entire country. For example, if we talk about India, multiple funding agencies such as DST, DBT, ICMR, etc., fund research in different domains. Instead, there could be a single central platform for antibiotic research funding (it could be named Department of Antibiotic Research or Indian Antibiotic Research Platform or any other), where only antibiotic research-based projects would be funded. In this way, the allocation of funds would be appropriately channelized, and duplication of efforts would be avoided. The unique platform should have different portals for early-, mid-, and late-stage funding. The research team working on early-stage research should enter into an early-stage portal and submit a proposal for funding. All country-wise platforms may be connected to a global platform. Moreover, similar to the clinical trial registry platform, there could be an antibiotic research registry platform, where it should be encouraged to register preclinical and clinical studies before execution. This may help in generating a centralized antibiotic research database that could make the antibiotic R&D status transparent.

In this review, we tried to cover the major push funding mechanisms. However, a lot more exists beyond these at various country levels. We apologize to the push funders for not comprehensively covering all the mechanisms, as it is too vast to cover all in a review. Needless to say, along with push incentives, equal attention is needed towards pull incentives to sustain the approved antibiotics in the market. Currently, more than two-thirds of the funding is towards push incentives which alone will not be able to suffice the pipeline. The discussion over pull incentives is out of the scope of this article, but replenishing the antibiotic R&D pipeline will not be possible without adequate pull incentives. Hence, there is a necessity to address the economic incentive needs of the developers; otherwise, the faintly appeared silver lining in the antibiotic research clouds may again darken.

## 5. Conclusions

Antibiotic resistance is a silent pandemic and a growing threat to public health. Revitalizing the antibiotic pipeline through financial assistance in the form of push funding and uniting the scientific community can bring back the lost art of discovery. Various push funding mechanisms in the last decade have tried to narrow the discovery void that occurred after the lucrative 1980s era of antibiotic development. However, despite numerous funding mechanisms, the pace of development is still slow, and the antibiotic market is unattractive for the big pharmaceuticals. Alongwith push funding, there is a need to incentivize antibiotic developers after regulatory approval to sustain the market.

The concern of exorbitant early-stage funding and little funding for SMEs struggling for mid- and late-stage developments are now being handled by several new mechanisms such as CARB-X, GARDP, ND4BB, etc. Further, to raise the voice of SMEs, the launch of the BEAM Alliance in 2015 by the EU is commendable. Another achievement is the foundation of the Global AMR R&D hub in 2018,which is helping to make the funding mechanisms and priorities transparent through its dynamic dashboard. Similar to the Global AMR R&D hub, a country-wise centralized database along with a central funding mechanism to appropriately channel the allocation of funds and to avoid efforts duplication can be considered for antibiotic research. Additionally, the formation of an antibiotic research registry platform can be considered, where it should be encouraged to register preclinical and clinical studies before execution to generate a centralized antibiotic research database.

The development of REVIVE platform by GARDP is another step in the right direction to bring the scientific community and experts in the antibiotic research field together, as many researchers in the field have retired, and a majority of the budding researchers are moving towards other lucrative fields, leading to little knowledge dissemination. REVIVE may revive back the scientific curiosity and vigor in the antibiotic R&D field. Now, the time has come to streamline the efforts and existing funding mechanisms in a coordinated and transparent way, along with a global collaboration to tackle this silently heading pandemic.

## Figures and Tables

**Table 1:** Summary of the major national and global push funding mechanisms adopted by the United States (US), European Union (EU), and United Kingdom (UK) to fund antibiotic research and development.

	Initiatives	Year	R&D Stage	Institutions	Countries
National Initiatives					
US					
	NIAID	2008	Early-to-late-stage	Research organizations, SMEs	US
	BARDA	2010	Majorly late-stage	SMEs, big pharmaceuticals	US, funds globally through CARB-X
	DOD	2012	Early-stage	Research organizations	US
	FDA	2014	Regulatory science research	Industries and research organizations	US
**EU**					
	IMI-ND4BB	2011	Early-stage to market	Industries and research organizations	EU member states and FP7-associated countries
	IDFF	2014	Late-stage	Industries and research organizations	28 EU member states and 17 countries associated with Horizon 2020
**UK**					
	NIHR	2008	Early-to-late-stage	Research organizations	UK (England-centric)
	MRC	2013	Majorly early-stage	Industries and research organizations	UK
**Global Initiatives**					
**US-based**					
	NIAID’s ARLG	2013	Late-stage	Industries and research organizations	Collaboration with 19 countries
	CARB-X	2016	Early-stage to Phase 1 trials	Industries and research organizations	Global collaboration
**EU based**					
	EDCTP	2003	Late-stage	Industries and research organizations	Europe-Africa initiative
	JPIAMR	2011	Early-stage	Research organizations	Collaboration with 29 member countries and European Commission
	GARDP	2016	Pipeline coordinator, Early-stage to market	Industries and research organizations	Global collaboration
**UK based**					
	Wellcome	2013	Early- to late-stage	Industries and research organizations	Global collaboration, Funds CARB-X and AMR Action Fund
	DHSC’s GAMRIF	2014	Majorly Early-stage	Industries and research organizations	Funds JPIAMR, CARB-X
	BEIS’s Newton Fund	2014	Early-stage	Research organizations	UK partnership with 16 countries inAsia, Africa, and Latin America
	BEIS’s GCRF	2015	Early- to late-stage	Industries and research organizations	UK partnership with developing countries
	DFID’s PDP	2017	Early- to late-stage	Non-profit partnerships	Collaboration with global organizations such as FIND, GARDP, etc.
**Pharmaceutical funding**					
	REPAIR Impact Fund	2018	Early-stage to Phase 1 trials	Start-ups, early-stage companies, and corporate spinouts	Europe and US
	AMR Action Fund	2020	Late-stage	Pharmaceutical companies	Global collaboration

NIAID: National Institute of Allergy and Infectious Diseases; BARDA: Biomedical Advanced Research and Development Authority; DOD: Department of Defense; FDA: Food and Drug Administration; SMEs: Small and medium size enterprises; FP7: EU’s Seventh Framework Programme; IMI-ND4BB: Innovative Medicine Initiative-New Drugs for Bad Bugs, IDFF: Innovfin Infectious Disease Facility; NIHR: National Institute for Health and Care Research; MRC: Medical Research Council; ARLG: Antibacterial Resistance Leadership Group; CARB-X: Combating Antibiotic-Resistant Bacteria Biopharmaceutical Accelerator; EDCTP: European and Developing Countries Clinical Trials Partnership; JPIAMR: Joint Programming Initiative on Antimicrobial Resistance; GARDP: Global Antibiotic Research and Development Partnership ;DHSC: Department for Health and Social Care; GAMRIF: Global AMR Innovation Fund; BEIS: Department for Business, Energy and Industrial Strategy; GCRF: Global Challenge Research Funds; DFID: Department for International Development; PDP: Product Development Partnerships; REPAIR: Replenishing and Enabling the Pipeline for Anti-Infective Resistance.

## Data Availability

Not applicable.

## Abbreviations

AHRC: Arts and Humanities Research Council, AMR: Antimicrobial Resistance, AMRFF: AMR Funders’ Forum, AR Isolate Bank: Antibiotic Resistance Isolate Bank, ARLG: Antibacterial Resistance Leadership Group, BARDA: Biomedical Advanced Research and Development Authority, BBSRC: Biotechnology & Biological Sciences Research Council, BEAM: Biotech companies from Europe innovating in Anti-Microbial resistance research, BEI: Biodefense and Emerging Infections Research, BEIS: Department for Business, Energy and Industrial Strategy, CARB: Combating Antibiotic-Resistant Bacteria, CARB-X: Combating Antibiotic Resistant Bacteria Biopharmaceutical Accelerator, CBRN: Chemical, Biological, Radiological And Nuclear, CDC: Center for Disease Control and Prevention, CDI: Clostridium difficile infection, CO-ADD: Community for Open Antimicrobial Drug Discovery, CRE: Carbapenem-resistant enterobacteriaceae, DFID: Department for International Development, DHHS: Department of Health and Human Services, DHSC: Department of Health and Social Care, DNDi: Drugs for Neglected Diseases initiative, DOD: Department of Defense, EC: European Commission, EDCTP: European & Developing Countries Clinical Trials Partnership, EFPIA: European Federation of Pharmaceutical Industries and Associations, EIC: European Innovation Council, ETP: European Technology Platform, EU: European Union, FDA: Food and Drug Administration, FIND: Foundation for Innovative New Diagnostics, FP7: EU’s Seventh Framework Programme, GAMRIF: Global AMR Innovation Fund, GARDP: Global Antibiotic Research and Development Partnership, GCRF: Global Challenge Research Funds, GH EDCTP3 JU: Global Health EDCTP3 Joint Undertaking, Global AMR R&D Hub: Global Antimicrobial Resistance (AMR) Research and Development (R&D) Hub, IDFF: Innovfin Infectious Disease Facility, IFPMA: International Federation of Pharmaceutical Manufacturers & Associations, IMI: Innovative Medicine Initiative, IMI-ND4BB: Innovative Medicine Initiative-New Drugs for Bad Bugs, IND: Investigational New Drug, JPIAMR: Joint Programming Initiative on Antimicrobial Resistance, MRC: Medical Research Council, MVP: Meningitis Vaccine Project, ND4BB: New Drugs for Bad Bugs, NHS: National Health Service, NIAID: National Institute of Allergy and Infectious Diseases, NIH: National Institute of Health, NIHR: National Institute for Health and Care Research, NPV: Net Present Value, ODA: Official Development Assistance, OTA: Other Transaction Authority, PAHPA: Pandemic and All-Hazards Preparedness Act, R&D: Research and Development, REPAIR: Replenishing and Enabling the Pipeline for Anti-Infective Resistance, SAP: Stewardship and Access Plan, SBIR: Small Business Innovative Research, SME:Small and medium size enterprises, STTR: Small Business Technology Transfer, TRL: Technology Readiness Levels, UK: United Kingdom, UKRI: UK Research and Innovation councils, US: United States of America, WHO: World Health Organization.
